# SMALL INTESTINAL BACTERIAL OVERGROWTH IN PEOPLE WITH CYSTIC FIBROSIS: SYSTEMATIC REVIEW

**DOI:** 10.1590/S0004-2803.24612024-110

**Published:** 2025-05-02

**Authors:** Maria Lidiane Lavor LANDIM, José Dirceu RIBEIRO, Daniela de Souza Paiva BORGLI, Danielle Rossana Queiroz Martins BONILHA, Elizete Aparecida LOMAZI, Maria de Fátima Correa Pimenta SERVIDONI

**Affiliations:** 1 Universidade Estadual de Campinas, Campinas, SP, Brasil.

**Keywords:** Cystic fibrosis, bacterial overgrowth, pancreatic insufficiency, steatorrhea, breath tests, Fibrose cística, supercrescimento bacteriano, insuficiência pancreática, esteatorreia, testes respiratórios

## Abstract

**Background:**

In patients with cystic fibrosis (pwCF) acid suppression therapy, gastrointestinal dysmotility, and post-operative bowel status, may predispose to the development of small intestinal bacterial overgrowth (SIBO). SIBO may continue to be present in the progression of the disease even on modulators. Breath testing is the most simple, non-invasive and available method for diagnosing SIBO. There are some divergencies over the operational procedures used to carry out and interpret breath tests in pwCF.

**Objective:**

We performed a systematic review of SIBO in pwCF to assess the methods used in breath tests and the existence of causal relationship between SIBO and following CF co-morbidities: liver disease, fat absorption, and eating disorders.

**Methods:**

We searched the PubMed, Cochrane Library, Embase, LILACS, MEDLINE, OpenGray, medRxiv, Google Scholar, and CAPES databases up to March 20, 2024. We selected clinical cohort and case-control studies to assess SIBO in cwCF. We selected studies that met the following criteria: (1) participants - children and adolescents diagnosed with CF; (2) intervention - assessment of SIBO using H_2_ and CH_4_ breath tests; (3) control - patients without SIBO; and (4) outcome - assessment of breath tests for SIBO diagnosis and the causal relationship between SIBO and CF co-morbidities. The PRISMA statement was used to report the search. QUADAS 2 tool was used for assessing the quality of each study methodology. The protocol for this review was registered in the Prospective Registration of Systematic Review Database (CRD42024503593).

**Results:**

The search strategy identified 279 studies. After screening titles and abstracts, 36 studies were selected for full-text review and 27 were excluded; nine studies involving 206 pwCFs were reviewed. All nine studies used H2 breath tests as a diagnostic method for SIBO, and five of them used a combined H2/CH4 test. There was no consistency in the timing of cessation of antibiotic therapy prior to testing. All patients performed the test after an overnight fast. A basal sample was collected prior to substrate (glucose or lactulose) ingestion, which ranged from 7 to 20 ppm. There was great variability between respiratory sample collection times, being times 0, 15, 30, 45, 60, 90, and 120 minutes the most used protocol. The methods for performing breath tests varied widely, making it difficult to reach conclusions on the role of SIBO as a co-morbidity in pwCF. There was no association between increased serum AST, ALT, and GGT levels and positive breath tests. There was no agreement regarding the role of SIBO and nutritional deficiency, but a reduction in fat absorption and the presence of hyporexia have been described under this condition.

**Conclusion:**

Data on assessment of SIBO in pwCF is limited by the small number of studies available, the lack of appropriate controls in some studies, and the varying test methodology and diagnostic cut-offs applied. Protocols to investigate and diagnosing SIBO in pwCF need to be developed.

## HIGHLIGHTS

• Primary studies on small intestinal bacterial overgrowth (SIBO) by using breath tests in patients with cystic fibrosis were analyzed.

• We selected and synthesized the data using the PRISMA methodology.

• Nine included studies described the prevalence of SIBO between 31.6 and 71%, higher than that of healthy controls (7 to 20%), however breath tests methodologies varied widely and were not comparable.

• Breath tests used in reviewed studies were methodologically different, and such differences make it difficult to draw conclusions about prevalence and causal relationships with co-morbidities presented by patients with CF.

## RESUMO

**Contexto:**

Em pacientes com fibrose cística (FC), a terapia de supressão ácida, dismotilidades gastrointestinais e o status pós-operatório de cirurgias intestinais podem predispor ao desenvolvimento de supercrescimento bacteriano do intestino delgado (SIBO). O SIBO pode continuar presente na progressão da doença, mesmo com o uso de moduladores. O teste respiratório é o método mais simples, não invasivo e amplamente disponível para diagnosticar SIBO. Contudo, há algumas divergências sobre os procedimentos operacionais usados para realizar e interpretar esses testes de pacientes com FC.

**Objetivo:**

Realizamos uma revisão sistemática do SIBO em pessoas com FC para avaliar os métodos usados nos testes respiratórios e a existência de relação causal entre SIBO e as seguintes comorbidades no paciente com FC: doença hepática, absorção de gordura e distúrbios nutricionais/alimentares.

**Métodos:**

Realizamos buscas nos bancos de dados PubMed, Cochrane Library, Embase, LILACS, MEDLINE, OpenGray, medRxiv, Google Scholar e CAPES incluindo artigos publicados até 20 de março de 2024. Selecionamos estudos clínicos de coorte e caso-controle para avaliar a SIBO em pacientes com FC. Selecionamos estudos que atendiam aos seguintes critérios: (1) participantes - crianças e adolescentes diagnosticados com FC; (2) intervenção - avaliação de SIBO usando testes respiratórios com dosagens de H2 ou CH4; (3) controle - pacientes sem SIBO; e (4) resultado - avaliação dos procedimentos operacionais de realização dos testes respiratórios para diagnóstico de SIBO e a relação causal entre SIBO e comorbidades em paciente com FC. A estratégia PRISMA foi usada para relatar a pesquisa. A ferramenta QUADAS 2 foi utilizada para avaliar qualidade da metodologia de cada estudo. O protocolo para essa revisão foi registrado no Prospective Registration of Systematic Review Database (CRD42024503593).

**Resultados:**

A estratégia de busca identificou 279 estudos. Após a triagem de títulos e resumos, 36 estudos foram selecionados para revisão de texto completo e 27 foram excluídos; nove estudos envolvendo 206 pacientes com FC foram revisados. Todos os nove estudos usaram testes respiratórios com H2 e cinco associaram H2/CH4 expirados. Não houve uniformidade quanto ao tempo de suspensão de antibioticoterapia previamente a realização dos testes. Todos os pacientes realizaram o teste em jejum. Uma amostra basal foi coletada antes da ingesta do substrato (glicose ou lactulose), variando de 7 a 20 ppm. Houve grande variabilidade entre o tempo de coleta das amostras respiratórias, sendo o protocolo de tempos 0, 15, 30, 45, 60, 90 e 120 minutos, o mais utilizado. Os métodos para a realização de testes respiratórios apresentaram grande variabilidade, dificultando a obtenção de conclusões sobre o papel do SIBO como uma comorbidade em pessoas com FC. Não houve associação entre o aumento dos níveis séricos de AST, ALT e GGT e testes respiratórios positivos. Não houve concordância com relação ao papel do SIBO na deficiência nutricional, mas uma redução na absorção de gordura e a presença de hiporexia foram descritas nessa condição.

**Conclusão:**

Os dados sobre a avaliação do SIBO em pessoas com FC são limitados pelo pequeno número de estudos disponíveis, pela falta de controles pareados e pela variação da metodologia dos testes diagnósticos aplicados. É necessário desenvolver protocolos para investigar e diagnosticar SIBO em pessoas com FC.

## INTRODUCTION

The term small intestinal bacterial overgrowth (SIBO) defines an excessive number in microorganisms in the small intestine^1^. In children, it can present with bloating and chronic abdominal pain, with or without diarrhea, nutrient deficiencies and weight loss^2^ ^,^ ^3^. SIBO is a common underlying diagnosis in children who clinically present with certain functional gastrointestinal disorders (FGDs) and stunted growth, and in children with a history of acid suppressive therapies, intestinal motility disorders, anatomical alterations, or impoverished conditions^2^. 

The diagnosis of SIBO can be defined by jejunal aspirate culture with a bacterial count greater than 10^5^ bacteria/ml of jejunal fluid, or by non-invasive methods such as breath tests (BTs)^4^ ^,^ ^5^. Glucose and lactulose breath testing have become more common in clinical practice as they are noninvasive, easily accessible, and have lower cost.

In patients with cystic fibrosis (pwCF) some conditions, such as acid suppression therapy, gastrointestinal dysmotility, and gastrointestinal anatomy, may predispose patients to the development of SIBO^3^ ^,^ ^6^. 

Combined measurement of hydrogen (H2) and methane (CH4)^7^ ^,^ ^8^ breath testing may be needed to confirm SIBO diagnosis in pwCF. Methane-producing colonic bacteria are more common in cystic fibrosis (CF) guts^2^ and may lead to false-negative H2 breath test results^9^. 

Despite recent advances in the treatment of CF with CFTR modulators^10^, observational studies have failed to demonstrate improvements in gastrointestinal symptoms such as pulmonary symptoms^11^.

Studies have reported the occurrence of SIBO in patients with CF as 30-50%, causing fat and carbohydrate malabsorption and subsequent weight loss. This diagnosis should be considered when gastrointestinal symptoms such as abdominal pain and/or bloating and steatorrhea persist despite the optimal use of pancreatic enzymes. SIBO may also be a risk factor for the development of non-alcoholic fatty liver disease (NAFLD) in children^12^ ^-^ ^15^.

This systematic review aimed to assess the methods used in breath tests in children with CF to diagnose SIBO and the causal relationship between SIBO and co-morbidities related to CF.

## METHODS

**FIGURE 1:**
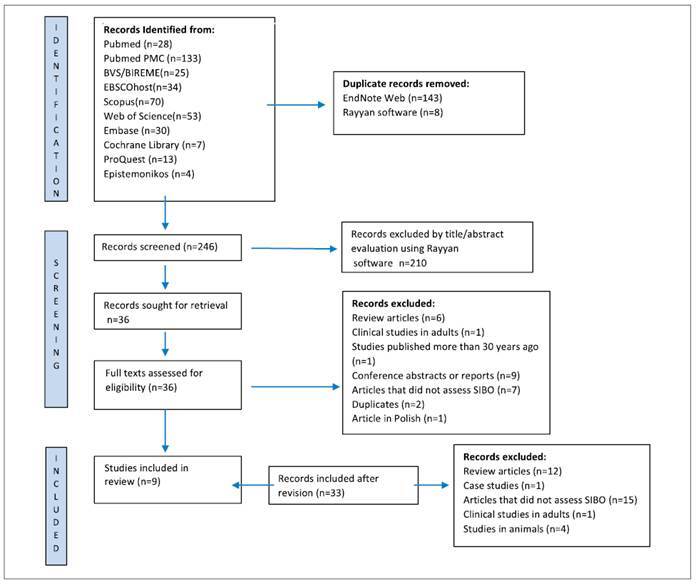
Preferred reporting items for a systematic review and meta-analysis flow chart.

This systematic review was structured in accordance with the Ministry of Health’s Preferred Reporting Items for a Systematic Review^16^ and is displayed in Figure 1 according to PRISMA (Preferred Reporting Items for a Systematic Review and Meta-analysis). The protocol for this review was registered in the Prospective Registration of Systematic Review Database (CRD42024503593).

### Data sources and research strategy

The following databases were serched: PubMed, PubMed PMC, BVS/BIREME, EBSCOhost, Scopus, Web of Science, Embase, Cochrane Library, ProQuest, and Epistemonikos. We used the search strategy: (Child OR Adolescent) AND “Cystic Fibrosis” AND “small intestinal bacterial overgrowth (SIBO) “ OR “small intestinal bacterial overgrowth” OR “Bacterial overgrowth” OR “contaminated small bowel syndrome” OR “SBBO (small bowel bacterial overgrowth) “ OR “SIBO” OR “SIBO syndrome” OR “small bowel bacterial overgrowth syndrome” OR “small bowel bacterial overgrowth” OR “small intestinal bacterial overgrowth” OR “small intestinal bacterial over-growth” OR “small intestinal bacterial overgrowth syndrome” OR “small intestinal bowel overgrowth” OR “small intestinal overgrowth” OR “small intestinal bacterial overgrowth” OR “upper gut bacterial overgrowth”. The search was updated on March 20, 2024. We checked the reference lists for additional article inclusion and no new articles were added.

### Eligibility and study selection

We selected studies that met the following criteria: (1) participants - children and adolescents diagnosed with CF; (2) intervention - assessment of SIBO using H_2_ and CH_4_ breath tests; (3) control - patients without SIBO; and (4) outcome - the role of SIBO in the management of eating disorders, fat absorption, liver disease, and use of medication. Review articles, animal research, conference abstracts or reports, clinical studies on adult populations, and case reports were excluded. Articles published in the past 30 years were screened, and language restrictions were imposed on one article written in Polish.

Two researchers (MF and ML) independently screened the selected studies by title and abstract using the Rayyan® software. When data was insufficient for evaluation based on the title/abstract alone, full-text articles were retrieved and evaluated. A third researcher (JD) assessed and resolved any disagreements between the first two researchers.

After reviewing the full text, articles that met the inclusion criteria were included in this review.

### Data extraction and quality assessment of individual studies

Data were extracted from the articles selected by one researcher (ML) and subsequently evaluated by another researcher (JD). The following data were extracted: year of publication, country of study, population and sample, design, diagnosis of SIBO by H_2_ and CH_4_ breath tests (basal value, collection time, dose of substrate, positivity criteria, medications in use during the test), prevalence of SIBO and its association with liver disease, nutritional deficiency, fat absorption, eating disorders, and use of antibiotics. Disagreements regarding data extraction were resolved by consensus.

Two researchers (ML and MF) assessed the quality of each study methodology individually using the Quality Assessment of Diagnostic Accuracy Studies 2 (QUADAS-2) tool. QUADAS-2 assesses the risk of bias in four key domains: patient selection, index test, reference standard, and flow and timing^17^. 

### Assessment summary

The accuracy measures included sensitivity and specificity. The *P*-value was evaluated to define whether the association between two variables could be a result of chance or inferred from the population.

### Summary of results

The original studies included in this systematic review did not contain quantitative convergent data. Therefore, it was not possible to perform a meta-analysis. The results will therefore be presented as a qualitative summary of the findings.

## RESULTS

The search strategy identified 279 studies. After evaluating titles and abstracts using the Rayyan® platform by two researchers, 36 studies were selected for full-text review. Twenty-seven records were excluded because they included review articles (n=6), clinical studies on adults (n=1), studies published over 30 years ago (n=1), conference abstracts or reports (n=9), articles that did not assess SIBO (n=7), duplicates (n=2), and article published in Polish (n=1) (FIGURE 1). 

The search on Google Scholar and grey literature (Google Scholar and CAPES) did not add studies to the list of references. No studies have been published by our group. Thus, nine studies involving at least 206 children diagnosed with CF were included in this systematic review.

### Characteristics of the studies

Table 1 and 1A summarizes the data from the nine included studies. Six studies were conducted in Europe: three in Poland^7^ ^,^ ^18^ ^,^ ^19^, one in Italy^9^, Spain^20^, and Germany^21^, two in the United States^3^ ^,^ ^14^, and one in Australia^22^. These articles have been published between 1998 and 2022. The sample of children with CF ranged from 10 to 79 participants. The studies included children of all ages, from infants to adolescents under 18 years of age. Some studies did not stratify the age groups under 18, making it impossible to accurately estimate the number of children included. Fridge et al. (2007) reported 18 pwCF under the age of 16 and Furnari et al. (2018) evaluated 32 pwCF under the age of 14, making it impossible to define patients aged 16-18 and 14-18, respectively. Two studies included adults and children, but did not stratify by age group, and it was not possible to define the population under 18^14^ ^,^ ^18^. All nine studies used H_2_ breath tests as a diagnostic method for SIBO, being that five of them used a combined and five of them used a combined H_2_/CH_4_ test. 

**TABLE 1:** Characteristics of studies and methodology to perform breath tests in children with cystic fibrosis.

Author, Country, Year	CF (N)	Participant age group	Genotypes	FEV1 %	Pancreatic insufficiency	Prevalence of SIBO in CF
		M = months y = years		(mean)		(%)
Lewindon et al.^22^, Australia, 1998	19	5 m - 9 y	NA	NA	NA	32
Fridge et al.^3^, USA, 2007	25	6-46 y (n =18 <16 y)	F508del/F508del (n=15)	>80	100%	56
			Heterozygous F508del (n=NA)			
			Other (n=NA)			
Infante et al.^20^, Spain, 2008	10	5-17 y	NA	NA	NA	50
Schneider et al.^21^, Germany, 2009	40	4-18 y	F508del/F508del (n=13)	99,4	93%	67.5
			Heterozygous F508del (n=19)			
			Other (n=8)			
Lisowska et al.^7^, Poland, 2009	62	5-17 y	F508del/F508del (n=32)	73	100%	37.1
			Heterozygous F508del (n=18)			
			Other (n=12)			
Lisowska et al.^19^, Poland, 2010	25	5-16 y	F508del/F508del (n=15)	88	100%	40
			Heterozygous F508del (n=9)			
			Other (n=1)			
Lisowska et al.^18^, Poland, 2011	26	12-32 y	F508del/F508del (n=12)	81.2	100%	100*
		(n<18 y non-available)	Heterozygous F508del (n=10)			
			Other (n=4)			
Furnari et al.^9^, Italy, 2018	79	9.2-36.9 y	NA	86	46-58.2%	31.6
		(n <14 y = 32)				
Gabel et al.^14^, USA, 2022	42	12-51 y	F508del/F508del (n=42)	84.9	NA**	71

* A sample of 100% pwCF and a diagnosis of SIBO (positive hydrogen-methane breath test) was taken by Lisowska et al(18). ** Probably 100% of patients have pancreatic insufficiency due to homozygous F508del mutation.

**TABLE 1A:** Continued

Author, year, country	Breath Test					
	Medication used	Basal level (ppm)	Sample collection time (min)	Dose	Positivity criteria	
					Hydrogen (H_2_)	Methane (CH_4_)
Lewindon et al.^22^, Australia, 1998	All children with CF were using ATB for prophylaxis or treatment	NA	Every 30 min for at least 210 min	Lactulose: 10 mL + 100mL of water	Rise of >10 ppm in two consecutive samples (30 min apart): “double peak”	
Fridge et al.^3^, USA, 2007	No ATB for 30 days; Use of oral tobramycin and azithromycin 3x/week	15	0, 5, 10, 15, 20, 25, 30, 40, 50, 60, 75, 90, 105, 120, 150 (15 samples)	Glucose: 1.5-2 g/kg (max 50-80g)	Fasting H_2_ >= 15ppm or rise of >= 10 over baseline during the test	2-fold increase over baseline
Infante et al.^20^, Spain, 2008	No ATB for 30 days	7	0, 10, 20, 30, 40, 50, 60, 80, 100, 120, 150	Glucose: 2 g/kg (max 80g)	Basal level >15 ppm or rise of >10 ppm above BL at any time during the test	
Schneider et al.^21^, Germany, 2009	No medication discontinuation	20	Every 30min for 3h	Glucose: 1 g/kg	Increase of BL by 20 ppm	
Lisowska et al.^7^, Poland, 2009	No ATB for 6 weeks	NA	0, 15, 30, 45, 60, 90, 120	Glucose: 1.5 g/kg (max 75 g)	Fasting H_2_ >= 20 ppm or a rise of >= 12 ppm over BL	Fasting >= 10 ppm or a rise >= 6 ppm over BL
Lisowska et al.^19^, Poland, 2010	No ATB for 6 weeks	NA	0, 15, 30, 45, 60, 90, 120	Glucose: 1.5 g/kg (max 75 g)	Fasting H_2_ >= 20ppm or rise of >= 12 ppm over BL	Rise of >= 6 ppm over BL
Lisowska et al.^18^ Poland, 2011	No ATB for 6 days	NA	0, 15, 30, 45, 60, 90, 120	Glucose: 1.5 g/kg (max 75g)	Rise of >= 20 ppm over BL	Rise of >= 12 ppm over BL
Furnari et al.^9^, Italy, 2018	No ATB for 30 days	12	Every 15min for 2h	Glucose: 2 g/kg (max 50 g)	Rise of >= 10 over BL	Rise of >= 12 over BL during the test
Gabel et al.^14^, USA, 2022	No oral, inhaled or IV ATB or systemic corticosteroids during 2 weeks	NA	0, 15, 30, 45, 60, 90, 120	Glucose: 1.75 g/kg (max 75g)	Fasting H_2_ > 15 ppm or a rise of >= 10 ppm above fasting levels	Fasting methane >= 10 ppm or a rise >= 6 over fasting levels

ALT, alanine aminotransferase; AST, aspartate aminotransferase; ATB, antibiotics; AZT, azithromycin; BL, baseline; BMI, body mass index; BT, breath test; CF, cystic fibrosis; FENIR, fecal near-infrared spectroscopy; FEV1: Forced expiratory volume in one second; GGT, gamma-glutamyl transferase; IV, intravenous; NA, not assessed; p, percentile; SIBO, small intestinal bacterial overgrowth.

Table 2 shows the Individual assessment of the quality of studies using QUADAS-2. The studies included in the review had a low risk of bias in most domains.

**TABLE 2:** Individual assessment of the quality of studies using QUADAS-2 (Quality Assessment of Diagnostic Accuracy Studies)^16^.

	Items	Lewindon et al., 1998	Fridge et al., 2007	Infante et al., 2008	Schneider et al., 2009	Lisowska et al., 2009	Lisowska et al., 2010	Lisowska et al., 2011	Furnari et al., 2018	Gabel et al., 2022
Domain 1: Patient Selection	Was a consecutive or random sample of patients enrolled?	Y	Y	Y	U	Y	Y	Y	Y	Y
	Was a case-control design avoided?	Y	Y	Y	Y	Y	Y	Y	Y	Y
	Did the study avoid inappropriate exclusions?	Y	Y	Y	Y	Y	Y	Y	Y	Y
	Could the selection of patients have introduced bias?	L	L	L	U	L	L	L	L	L
	Applicability: Are there concerns that the included patients and parameters do not match the review question?	L	L	L	L	L	L	L	L	L
Domain 2: Index Test	Were the index test results interpreted without knowledge of the results of the reference standard?	Y	Y	Y	Y	Y	Y	Y	Y	Y
	If a threshold was used, was it pre-specified?	Y	Y	Y	Y	Y	Y	Y	Y	Y
	Could the conduct or interpretation of the index test have introduced bias?	L	L	L	L	L	L	L	L	L
	Applicability: Are there concerns that the index test, its conduct, or interpretation differ from the review question?	L	L	L	L	L	L	L	L	L
Domain 3: Reference Standard	Is the reference standard likely to correctly classify the target condition?	Y	Y	Y	Y	Y	Y	Y	Y	Y
	Were the reference standard results interpreted without knowledge of the results of the index test?	Y	Y	Y	Y	Y	Y	Y	Y	Y
	Could the reference standard, its conduct, or its interpretation have introduced bias?	L	L	L	L	L	L	L	L	L
	Are there concerns that the target condition as defined by the reference standard does not match the review question?	L	L	L	L	L	L	L	L	L
Domain 4: Flow and Timing	Was there an appropriate interval between index test(s) and reference standard?	Y	Y	Y	Y	Y	Y	Y	Y	Y
	Did all patients receive the same reference standard?	Y	N	Y	Y	N	U	Y	Y	Y
	Were all patients included in the analysis?	Y	Y	Y	Y	Y	Y	Y	Y	Y
	Could the patient flow have introduced bias?	L	H	L	L	H	U	L	L	L

Y: yes; N: no; U: unclear; L: low risk of bias; H; high risk of bias.

### Breath tests for diagnosing SIBO in CF

The most used device for performing breath tests was the Quintron Microlyser DP^3^ ^,^ ^7^ ^,^ ^9^ ^,^ ^18^ ^,^ ^19^ ^,^ ^20^, followed by the electrochemical analyzer (Stimotron Medical, Wendelstein, Germany)^21^, and two studies did not describe it^14^ ^,^ ^22^.

Recommendations for discontinuation of antibiotics prior to testing ranged from 2 weeks^14^, 4 weeks (except for tobramycin and azitromycin three times weekly)^3^ ^,^ ^9^ ^,^ ^20^, and 6 weeks^7^ ^,^ ^18^ ^,^ ^19^. Some patients underwent breath testing while receiving antibiotics for prophylaxis or treatment^21^ ^,^ ^22^.

All patients completed the test after fasting and a basal sample was collected before substrate ingestion. This rate ranged from 7 to 20 ppm and no study has established a minimum limit as a criterion for performing the test.

The studies used glucose at a dose ranging from 1 to 2 g/kg (maximum dose of 50-80 g) as a substrate^3,7,9,14,18,19, 20,21^ and only one used lactulose at a pre-established dose of 10 mL^22^. Most studies have shown similar doses of glucose substrates used in breath tests.

There was great variability between respiratory sample collection times, being times 0, 15, 30, 45, 60, 90, and 120 minutes the most used protocol^7^ ^,^ ^14^ ^,^ ^18^ ^,^ ^19^.

Table 1 shows lack of uniformity in the positivity criteria, with variations between studies from the same health unit.

### Prevalence of SIBO

The prevalence of SIBO among patients diagnosed with CF varied from 31.6 to 71%, higher than that from controls non-CF (7-20%). A sample of 100% pwCF and a diagnosis of SIBO (positive hydrogen-methane breath test) was taken by Lisowska et al^18^.

### Liver disease

Only one study evaluated the association of elevated AST, ALT and GGT with positive BT for SIBO in patients with CF^3^.

### Nutritional deficiency/fat absorption

A positive association between positive BT for SIBO and low weight/BMI in children with CF has been reported^9^ ^,^ ^14^, but not confirmed in all studies^3^ ^,^ ^19^ ^,^ ^21^. SIBO was also associated with pancreatic insufficiency, steatorrhea, and carbohydrate malabsorption through H_2_ exhalation tests (lactose/sucrose malabsorption) and evaluation of fecal samples by fecal near infrared spectroscopy (FENIR)^20, 22^.

Breath testing with 13C-labeled mixed triglyceride reported that oral antibiotic therapy in CF and SIBO patients with pulmonary exacerbations resulted in increased fat digestion and absorption^18^.

### Eating disorders

Symptoms was available in one study, using a self-reported questionnaire that assessed seven variables: diarrhea, upper and lower abdominal pain, abdominal swelling, bloating, constipation, and hyporexia. Patients gave each symptom a score from 0 (no symptoms) to 3 (severe symptoms); patients under 14 years of age were assisted by their parents or tutors. Hyporexia was the only statistically significant symptom associated with positive BT results for SIBO^9^.

### Use of antibiotics

An increased risk of SIBO in patients using azithromycin three times a week was found in one study^3^, but not confirmed in another study that found no association between continuous use of azithromycin or inhaled tobramycin and SIBO^14^.

### Other risk factors assessed

There was no consensus regarding the positive association between using inhaled ipratropium and positive BTs for SIBO^3^ ^,^ ^9^. No association was observed between SIBO and the use of proton pump inhibitors^14^ ^,^ ^21^, increased fecal calprotectin^19^, sex, or diabetes mellitus^21^. Daily use of laxatives (PEG 3350 or docusate sodium) was associated with a decreased risk of positive BTs^3^. Patients with CF and SIBO did not show a longer intestinal transit time than those without SIBO^22^.

## DISCUSSION

Breath tests are widely used to diagnose SIBO in cwCF, but the lack of standardization in the protocols used makes it difficult to compare results from different centers. A standard protocol should include specific guidelines for pretest diet, medication restrictions, substrate dosage, sampling time, and diagnostic criteria.

A peculiar condition in cwCF is the frequent need of antibiotics, used to treat CF comorbidities what may reduce the sensitivity of BTs to detect SIBO^18^. However, elevated fasting H2 breath test values were found in patients without antibiotics (53%), and much higher values were found in children with acute (82%) and chronic (75%) antibiotic use^21^.

Some articles in this review used BTs combining H2 and CH4 measurements to diagnose SIBO^3^ ^,^ ^7^ ^,^ ^9^ ^,^ ^18^ ^,^ ^19^, with CH4 measurement leading to the diagnosis in 16%^9^ and 30.4%^7^ of patients with a negative H2 test. The most effective method for assessing SIBO in children with CF has not yet been established, but H2 measurements alone may not be sufficient to provide an accurate diagnosis of SIBO in some patients. Methane-producing bacteria are more common in CF guts^2^ and may lead to false-negative H2 test results^8^.

The mechanisms linking liver disease (non-alcoholic fatty liver disease) to SIBO are not well understood but may involve increased intestinal permeability with the release of bacterial toxins into the bloodstream, triggering an inflammatory state^23^ ^,^ ^24^. Fridge et al. (2007) were the only authors who identified elevated AST, ALT, and GGT levels associated with positive BTs for SIBO, even though there was no significant difference between groups^3^.

The reported prevalence of SIBO in cwCF ranged from 31.6 to 71%. SIBO was more prevalent in patients with pancreatic insufficiency, steatorrhea, and carbohydrate malabsorption^9^ ^,^ ^18^ ^,^ ^20^, with subsequent difficulty in gaining weight and poor growth^9^ ^,^ ^14^. A positive correlation has been found between low BMI and positive BT independent of the presence of pancreatic insufficiency^9^.

Frequent use of antibiotics is regularly associated with the presence of SIBO, but studies have disagreed in proving this association in children with CF^3^ ^,^ ^14^. Oral antibiotic therapy in CF patients with SIBO increases fat digestion and absorption^18^, which may predict SIBO’s role in exacerbating steatorrhea and pancreatic insufficiency in these patients.

The only study evaluating treatment of SIBO included 13 patients (56.5%) in the rifaximin group and 10 patients (43.5%) in the no treatment control group. The SIBO eradication rate was 9/10 (90%) in the rifaximin group and 2/6 (33.3%) in the control group. An improvement in gastrointestinal symptoms was observed in the rifaximin group^9^.

Until the first decade of this century, the main strategy for treating CF was to target the consequences of the basic defect. More recently, a novel and highly effective therapy has become available with CFTR modulators, which can improve the function of the CFTR protein^10^. These agents have improved the quality of life and increased the life expectancy of patients with CF; however, observational studies have shown that the effect of modulators on gastrointestinal symptoms is less than that on pulmonary symptoms^11^. 

Recent studies report positive effects of CFTR modulators on pH levels and intestinal bicarbonate secretion, as well as improvements in inflammation, GERD, and dysbiosis-related symptoms^24^. CFTR dysfunction and its consequences may predispose to SIBO, but the only study that examined the modulation of CFTR and SIBO found no significant changes in the hydrogen breath test, despite a small sample size and short duration (one month)^14^ ^,^ ^25^.

Children with CF had a higher risk of developing SIBO than healthy controls. According to Dorsey and Gonska^26^, the pathophysiological basis of gastrointestinal symptoms in CF can be understood as the concomitant effect of three phenomena resulting from the defect of secretion of chloride and bicarbonate by enterocytes: inflammation, dysbiosis and dysmotility. These factors are attributed to the origin of symptoms that correspond to the clinical expression of several nosological entities that affect the intestine of CF patients, from the Meconium ileum to the increased risk for the development of intestinal neoplasms and including SIBO^27^ ^,^ ^28^. However, prevalence studies are based on tests that are not comparable and have operational procedures that do not consider the therapeutic needs of patients with CF, making it difficult to draw conclusions about the actual role of SIBO in the management of patients with CF.

In addition, we believe that further studies should be conducted to determine the role of SIBO in liver changes, pancreatic insufficiency, and nutritional deficiencies in patients with CF.

## CONCLUSION

We conclude that there are no uniform criteria for laboratory diagnosis of SIBO in cwCF. Criteria for the diagnosis and management of SIBO in these patients need to be established.

Prevalence studies are based on tests that are not comparable, making it difficult to draw conclusions regarding the true role of SIBO in the management of patients with CF. 

In addition, we believe that studies should be conducted to define the role of SIBO in liver changes, pancreatic insufficiency, and nutritional deficiencies in cwCF as well as to standardize BT protocols to compare data from different samples.
